# Draft genome sequence of *Legionella pneumophila* strains isolated from engineered water systems in Brazil

**DOI:** 10.1128/mra.01332-25

**Published:** 2026-04-20

**Authors:** Dândrea Driely de Melo Ferrari, Edson Machado, Emilyn Costa Conceição, Abhinav Sharma, Solange Lima, Karla Valéria Batista Lima, Raquel Lima de Figueiredo Teixeira, Harrison Magdinier Gomes, Philip Suffys

**Affiliations:** 1 Laboratory of Molecular Biology Applied to Mycobacteria, Oswaldo Cruz Institute, Oswaldo Cruz Foundation, Rio de Janeiro, Brazil; 2 Program in Clinical Research and Infectious Diseases, Evandro Chagas National Institute of Infectious Diseases, Oswaldo Cruz Foundation 663758 , Rio de Janeiro, Manguinhos, Brazil; 3 South African Medical Research Council Centre for Tuberculosis Research, Division of Molecular Biology and Human Genetics, Faculty of Medicine and Health Sciences, Stellenbosch University, Cape Town, South Africa; 4 Centre for Epidemic Response and Innovation (CERI), School of Data Science and Computational Thinking, Stellenbosch University, Cape Town, Western Cape, South Africa; 5 Conforlab Engenharia Ambiental, São Paulo, Brazil; 6 Bacteriology and Mycology Section, Evandro Chagas Institute 89124, Ananindeua, Pará, Brazil; Portland State University, Portland, Oregon, United States

**Keywords:** *Legionella pneumophila*

## Abstract

We report the draft genome sequence of 12 isolates of *Legionella pneumophila* subsp. *pneumophila* from Brazil. The genomes have a GC content of approximately 38%, genome sizes ranging from 3.20 to 4.09 Mb, and 2,845–3,615 predicted coding sequences.

## ANNOUNCEMENT

*Legionella pneumophila* is a ubiquitous environmental bacterium belonging to the Legionellaceae family. It is a small, gram-negative, strictly aerobic bacillus that does not produce spores or possess a capsule (1). Human infection occurs through the inhalation of aerosolized particles containing the pathogen, typically associated with engineered water systems. However, person-to-person transmission of Legionnaires’ disease has also been reported (2). The clinical spectrum of disease comprises two well-defined syndromes: Pontiac fever, an acute, self-limiting influenza-like illness, and Legionnaires’ disease, a severe pneumonia associated with significant morbidity and mortality (3). We report here the draft genome sequence of 12 strains of *L. pneumophila*, isolated as part of a surveillance study in Brazil, from nine hotels, a laboratory, and a shopping mall in eight Brazilian states (Table 1).

**TABLE 1:** Information on sequenced *Legionella* sp. isolates^*b*^

Sample	Year	Source	Local	City	State	GenBank accession no.	SRA	Organism	Contigs	Bases	Genes	% GC	N50	L50	Completeness	Contamination
ISHP06	2015	Drinking fountain	Hotel	Belo Horizonte	MG	JBPZJB000000000	SRR34759054	*L. pneumophila* subsp. *pneumophila*	64	3,551,892	3,214	38	116,016	10	100	0.19
ISHP07	2015	Faucet	Hotel	Porto Alegre	RS	JAJCCN000000000	SRR16377044	*L. pneumophila* subsp. *pneumophila*	52	3,350,592	3,038	38	145,133	8	100	0.38
ISHP03	2015	Faucet	Hotel	Vera Cruz	BA	JAJCCM000000000	SRR16377045	*L. pneumophila* subsp. *pneumophila*	106	3,219,274	2,897	38	64,772	15	100	0.38
18HP16	2018	Faucet	Hotel	Belo Horizonte	MG	JBPZJC000000000	SRR34759053	*L. pneumophila* subsp. *pneumophila*	58	3,554,197	3,212	38	137,947	9	100	0.19
18HP14	2018	Shower	Hotel	Rio de Janeiro	RJ	JBRKJP000000000	SRR16377043	*L. pneumophila* subsp. *pneumophila*	68	3,299,477	2,974	38	132,763	12	100	0.19
18SI20	2018	Cooling tower	Mall	São Paulo	SP	JBPZJD000000000	SRR34759052	*L. pneumophila* subsp. *pneumophila*	107	3,783,046	3,459	38	108,431	10	100	0.38
21121	2019	Faucet	Hotel^*a*^	Paranaguá	PR	JBPZJE000000000	SRR34759051	*L. pneumophila* subsp. *pneumophila*	30	3,276,353	2,962	38	346,681	4	100	0.19
21123	2019	Faucet	Hotel^*a*^	Paranaguá	PR	JBPZJF000000000	SRR34759050	*L. pneumophila* subsp. *pneumophila*	31	3,256,285	2,940	38	225,298	4	100	0.19
36803	2019	Faucet	Hotel	Aracaju	SE	JBPZJG000000000	SRR34759049	*L. pneumophila* subsp. *pneumophila*	61	3,442,241	3,111	38	133,048	9	100	0.19
37065	2019	Faucet	Hotel	Recife	PE	JBPZJI000000000	SRR34759047	*L. pneumophila* subsp. *pneumophila*	55	3,482,255	3,145	38	190,718	9	100	0.19
20935	2019	Shower	Hotel	Foz do Iguaçu	PR	JAJCCL000000000	SRR16377047	*L. pneumophila* subsp. *pneumophila*	35	3,361,576	3,021	38	174,743	6	100	0.19

^*a*^ Samples belong to the same hotel. CheckM estimates genome completeness and contamination based on the presence or absence of marker genes.

^*b*^ BA, Bahia; % GC, GC content; L50, the smallest number of contigs accounting for 50% of the total assembly length; MG, Minas Gerais; N50, the length of the shortest contig within this subset; PE, Pernambuco; PR, Paraná; RJ, Rio de Janeiro; RS, Rio Grande do Suk; SE, Sergipe; SP, São Paulo.

The bacteria were isolated in accordance with ISO 11731:2017 by Conforlab and sent to our laboratory, where a fresh culture prepared by inoculation in a plate with BCYE medium (Sigma-Aldrich, São Paulo, Brazil) at 37°C and 5% of CO_2_ up to 14 days. A loop of wet bacterial mass was then submitted to nucleic acid extraction in parallel with adding a similar amount to BYE broth with glycerol for long-term storage in liquid nitrogen.

Genomic DNA was extracted using the method described by Jahan et al. (4), including cell lysis using proteinase K followed by DNA purification using cetyltrimethylammonium bromide, isoamyl-chloroform, and isopropanol precipitation. We determined DNA quantity and quality using the Qubit Double-Strand DNA Broad Range Assay Kit (Thermo Fisher Scientific, Waltham, USA). For whole-genome sequencing, a library was prepared using the Nextera XT DNA Library Preparation Kit (Illumina, San Diego, USA) and after quality control using the 2100 Bioanalyzer (Agilent Technologies, Santa Clara, United States) with libraries requiring a minimum of 30 ng of total DNA, sequenced on a NextSeq 500 platform (Illumina), using the NextSeq 500/550 High Output 300 Cycles Kit, generating 2 × 150 paired-end fragments.

Quality control and trimming of the reads was done using Trimmomatic v0.39 (5), with the following parameters: LEADING:3, TRAILING:3, SLIDINGWINDOW:4:20, and MINLEN:40. Read quality was verified using FastQC v0.12.1 (6), and genomes were *de novo* assembled using SPAdes v3.15.4 (7). We checked the quality of the genomes with CheckM v1.2.2 (8), and the annotation was performed by the NCBI Prokaryotic Genome Annotation Pipeline (PGAP) v6.10 (9). Default parameters were used for all software unless otherwise specified. The characteristics of the assembled genomes are summarized in Table 1. Briefly, genome size ranged from 3.20 to 4.09 Mb; GC content was 38%; the number of contigs ranged from 31 to 107; and annotation predicted between 2,845 and 3,615 coding sequences.

To our knowledge, these are the first genomes from *L. pneumophila* isolated in Brazil. These data will allow better analysis of genetic variability of environmental *Legionella* strains in the country, which was previously only available through sequence typing (10), and will be the basis of more detailed genome analysis.

## Data Availability

The draft genome sequence of *L. pneumophila* strains has been deposited at GenBank. The raw reads are available in the Sequence Read Archive under the studies SRP604359 and SRP341755, associated with BioProject numbers PRJNA1297308 and PRJNA772000, respectively.
